# Silica Nanoparticles as a Probable Anti-Oomycete Compound Against Downy Mildew, and Yield and Quality Enhancer in Grapevines: Field Evaluation, Molecular, Physiological, Ultrastructural, and Toxicity Investigations

**DOI:** 10.3389/fpls.2021.763365

**Published:** 2021-10-28

**Authors:** Younes M. Rashad, Hany H. A. El-Sharkawy, Bassam E. A. Belal, Elsayed S. Abdel Razik, Doaa A. Galilah

**Affiliations:** ^1^Plant Protection and Biomolecular Diagnosis Department, Arid Lands Cultivation Research Institute, City of Scientific Research and Technological Applications (SRTA-City), New Borg El-Arab City, Egypt; ^2^Department of Mycology Research and Plant Disease Survey, Plant Pathology Research Institute, Agricultural Research Center, Giza, Egypt; ^3^Viticulture Department, Horticulture Research Institute, Agricultural Research Center, Giza, Egypt; ^4^Botany Department, Faculty of Science, Mansoura University, Mansoura, Egypt

**Keywords:** downy mildew, grapevine, plant resistance, *Plasmopara viticola*, SiNPs

## Abstract

Downy mildew is the most destructive disease of grapevines in the regions of relatively warm and humid climate causing up to 50% yield losses. Application of silicon- (Si-) based products have been extensively studied against various oomycete, fungal, bacterial, and viral plant diseases, but studies on Si application in their nanosize are limited. In this study, the field application of silica nanoparticles (SiNPs) on Thompson Seedless grapevines (H4 strain) infected with downy mildew was evaluated. In addition, molecular, physiological, ultrastructural, and toxicity investigations were also conducted. The obtained results revealed that spraying of grapevines with SiNPs at 150 ppm significantly overexpressed the transcription factor jasmonate and ethylene-responsive factor 3 recording 8.7-fold, and the defense-related genes β-1,3-glucanase (11-fold), peroxidase (10.7-fold) pathogenesis-related-protein 1 (10.6-fold), and chitinase (6.5-fold). Moreover, a reduction up to 81.5% in the disease severity was achieved in response to this treatment. Shoot length and yield per grapevine were considerably enhanced recording up to 26.3 and 23.7% increase, respectively. The berries quality was also improved. Furthermore, this treatment led to an enhancement in the photosynthetic pigments, induction of phenolic and ascorbic acid contents, an increase in the activity of peroxidase and polyphenol oxidase enzymes, and a reduction in the cellular electrolyte leakage, lipid peroxidation, and H_2_O_2_ content. Scanning electron microscopy observations showed an increase up to 86.6% in the number of closed stomata and a reduction up to 55% in the average stomatal pore area in response to this treatment. Observations of the transmission electron microscopy showed ultrastructural alterations in the cells of a grapevine leaf due to the infection with downy mildew, including plasmolysis and disruption of the cellular components, abnormal chloroplasts, and thickening of the cell wall and cell membrane. These abnormal alterations were reduced in response to SiNPs spray. In contrast, this study also showed that this treatment had considerable cytotoxic and genotoxic effects at this direct dose/concentration. So, additional investigations to determine the SiNPs residue in the produced edible plant parts are urgently needed. In addition, the pre-harvest interval, toxicity index, and risk assessment should be evaluated before any recommendation for use.

## Introduction

Grapevine (*Vitis vinifera* L.) is one of the most important commercial fruit crops grown worldwide for its edible berries, fresh or dry (raisins), or for the production of wine and juice. The grapevine plant is a woody, perennial, climbing, and vigorous vine belonging to the family Vitaceae. Globally, the grapevine is ranked fifth among the top produced fruits next to bananas, watermelons, apples, and oranges with a total worldwide production of more than 77 million tons (FAOSTAT, 2021). Thompson seedless grapevine (H4 strain) has received extensive attention in the recent few years due to its characteristic yield production, but the problem of this cultivar is its high susceptibility to infection with downy mildew.

Downy mildew, caused by *Plasmopara viticola* (Berk. and M. A. Curtis) Berl. and De Toni, is the most destructive disease of grapevines in the regions of relatively warm and humid climate. This obligate biotrophic oomycete (family: Peronosporaceae) attacks grapevines and severely reduces their growth and yield causing up to 50% losses (Gessler et al., 2011). Oospores (sexual spores) of the pathogen can survive up to 10 years in the leaf litter or field soil to the next season(s) and be dispersed to healthy plants by rain splashes or wind. Early symptoms include discoloration (brown-oily spotting) and necrosis on the shoot system, and later under favorable conditions, sporangia and sporangiophores of the pathogen can be seen as a white coat on the downside of the leaves and the outer surface of the stem and berries (Carisse, 2016). Copper-based anti-oomycete fungicides and other synthetic agrochemicals, such as fosetyl-aluminum, metalaxyl, and mandipropamid are available and still commonly used today for the control of downy mildew disease, but the improper and frequent use of these anti-oomycete fungicides led to evolution of the pathogen resistance to many anti-oomycete fungicides (Massi et al., 2021). In addition, accumulation of copper and other anti-oomycete fungicides in the soil represents environmental risk on plants and non-targeted soil microbiota (Bavaresco et al., 2019). Nowadays, the demand for new anti-oomycete fungicides is highly increased to overcome the risk of the recently appeared fungicide resistance in the downy mildew pathogen.

Silicon (Si) is the main component of the Earth’s soil constituting about 70% of its mass. Although Si is not recognized as one of the essential elements for plants, it constitutes from 0.1 to 10% of the plant dry weights, depending on the plant species, and has different beneficial influences on plant development and production especially under biotic and abiotic stresses (Luyckx et al., 2017b). The role of Si in triggering the plant resistance against different fungal and oomycete diseases, such as powdery mildew, septoria, stalk rot, fusarium wilt, leaf blast, and downy mildew, has been demonstrated (Vivancos et al., 2015; Jadhao and Rout, 2020; Kaur et al., 2021). The proposed defensive mechanisms include activation of the defense-related enzymes, accumulation of antifungal and anti-oomycete compounds, regulating signal pathways, an upregulation of the defense-related genes, and controlling of the phytohormones homeostasis (Wang et al., 2017). In addition, deposition of Si in plant tissues, especially the epidermal cells acts as a physical barrier to prevent pathogen penetration and to reduce the cell susceptibility to the enzymatic degradation during the pathogen invasion (Van Bockhaven et al., 2013). Moreover, Si fertilization has positive roles in reducing plant damages caused by grazing animals and insects (Alhousari and Greger, 2018), regulating the nutrient uptake (Luyckx et al., 2017b), mitigating metal toxicity on plant growth (Bhat et al., 2019), ameliorating plant development under drought stress (Bukhari et al., 2020), and alleviating salt stress (Guntzer et al., 2012). The utilization of nanotechnology to develop new sustainable strategies in agronomy is acquiring a high significance in the last years. However, uses of nanosized materials in agronomy are restricted by the cytotoxicity and genotoxicity they inflict upon the plants and their consumers. In this regard, impacts of silica nanoparticles (SiNPs) on plant physiology, which ranged from positive to negative effects, have been studied (Le et al., 2014; Suriyaprabha et al., 2014). In general, plant responses to nanoparticles (NPs) are dependent on many factors, including their size, shape, concentration, and utilization method (Rastogi et al., 2017). In this study, we aimed to (1) evaluate the field application of SiNPs on the grapevine resistance to downy mildew at pathological, biochemical, ultrastructural, and molecular levels, (2) investigate their impacts on the vegetative growth parameters, as well as yield and its quality parameters, and (3) study their cytotoxic and genotoxic impacts at the applied dose/concentration.

## Materials and Methods

### Silica Nanoparticles and Grapevine Cultivar

Amorphous SiNPs (Nanogate^®^, Cairo Governorate, Egypt) used in this study were spherical and had a particle size of 20 ± 4 nm. Grapevines of Thompson seedless (H4 strain) cultivar grafted on freedom rootstock were used in the field experiment.

### Field Experiment

The field experiment was conducted at the vineyard of El-Baramon Experimental Farm, Horticultural Research Institute, Mansoura, Egypt. About 60 grapevines, 6-years-old, grown in clay soil under a surface irrigation system were chosen as uniform in vigor as possible for this experiment. The physical and chemical properties of the used soil were as follows: texture (clay), organic matter content (1.92%), pH (7.9), electrical conductivity (0.68 mmhos cm^–1^), calcium carbonate content (1.78%), nitrogen content (31.4 ppm), phosphorus content (12.98 ppm), and potassium content (318.2 ppm). The grapevines were spaced at 2 × 3 m, and trellised with a Spanish baron system. During the 1st week of January in each experimental season, the tested grapevines were cane-pruned to 112 buds/vine (8 canes with 12 eyes and 8 spurs with 2 eyes). All grapevines received the same cultural management practices recommended by the Ministry of Agriculture. The grapevines, naturally infected with downy mildew, were separately sprayed with an aqueous solution of SiNPs at the rates of 50,100, and 150 ppm (1 L/grapevine) four times at 15 days from a bud burst (beginning of the vegetative growth stage), 45 days from a bud burst (before the bloom stage), 60 days from a bud burst (the bloom stage), and 85 days from a bud burst (2 weeks after the fruit set stage). The chemical anti-oomycete fungicide (Rent, Azoxystrobin + Dimethomorph, 80% WG) (Starchem Co., Cairo, Egypt) was applied as a positive control treatment at the rate of 0.3 g L^–1^. Grapevines sprayed with tap water were used as a negative control treatment. The experiment was composed of five treatments arranged in a randomized completely block design, each treatment was replicated three times and each replicate included four grapevines. All treatments were adjusted to 24 clusters/grapevine after the complete set of berries. The experiment was repeated over two successive seasons: the first was from April to July 2020, and the second was from April to July 2021. The weather conditions during the experiment period were as follows: average air temperature (18–28°C), relative humidity (50–58%), rainfall (0–4 mm), and daily sunshine hours (10.7–11.8 h).

### Time-Course Analysis of Gene Expression

Grapevine leaves were sampled at different intervals before the appearance of symptoms [1, 3, and 7 days post the first spray (dps1)] and after the appearance of symptoms [1, 3, and 7 days post the second spray (dps2)]. For messenger RNA (mRNA) extraction, the RNeasy Mini kit (Qiagen, Hilden, Germany) was used according to the instructions of the manufacturer.

Complementary DNA (cDNA) synthesis was carried out in a reaction mixture (20 μl) containing 10.8 μl RNase free water, 2.5 μl 10× buffer with MgCl_2_, 1 μl oligo (dT) primer (10 pmol μl^–1^), 2.5 μl deoxynucleotide triphosphates (dNTPs) (10 mM), 3 μl RNA (30 ng), and 0.2 μl reverse transcriptase enzyme (New England Biolabs, Frankfurt am Main, Germany). The PCR was done using a SureCycler 8800 (Agilent, Santa Clara, CA, United States) at 42°C for 2 h, and then at 70°C for 5 min, the product was stored at −80°C.

The quantitative Real-Time PCR (qPCR) reaction was made up of 1 μl cDNA, 10 μl 2xSYBR^®^ Green RT Mix (Bioloine, Germany), 1 μl of each forward and reverse primer (10 pmol μl^–1^), and 7 μl RNase free water. Sequences of the primers used in this investigation are presented in Table 1. The qPCR program was carried out as follows, 1 cycle at 95°C for 5 min, 40 cycles (95°C for 30 s, 56°C for 30 s, and 72°C for 30 s) using a Rotor-Gene-6000-system (Qiagen, Valencia, CA, United States). *β*-actin and elongation factor 1-α (*EF1-α*) were used as reference genes for their relevance and stability in a grapevine. The relative expression of the tested genes was calculated using the comparative C_*T*_ method (2^–ΔΔCT^) (Schmittgen and Livak, 2008). For each sample, triplicate biological and technical replications were done.

**TABLE 1:** Primer sequences of the genes studied in the qPCR.

Gene description	Abbrev.	Amplicon length	Annealing temperature (°C)	Melting temperature (°C)	Amplification efficiency	Accession no.	Sequence (5′–3′)
Jasmonate and ethylene-responsive factor 3	*JERF3*-F *JERF3*-R	101 bp	56	63	1.82	AY383630	GCCATTTGCCTTCTCTGCTTC GCAGCAGCATCCTTGTCTGA
Pathogenesis-related-protein 1	*PR1*-F *PR1*-R	90 bp	56	62	1.92	M69247	ACTTGGCATCCCGAGCACAA CTCGGACACCCACAATTGCA
Chitinase class II	*CHI II*-F *CHI II*-R	86 bp	56	63	1.01	U30465	GCGTTGTGGTTCTGGATGACA CAGCGGCAGAATCAGCAACA
*β*-1,3-glucanase	*Glu*-F *Glu*-R	124 bp	56	62	1.91	M80604	TTTCGATGCCCTTGTGGATTC GGCCAACCACTTTCCGATAC
Peroxidase	*POD*-F *POD*-R	111 bp	56	62	1.87	X94943	CCTTGTTGGTGGGCACACAA GGCCACCAGTGGAGTTGAAA
*β*-actin	*β*-actin-F *β*-actin-R	118 bp	56	62	2.0		GTGGGCCGCTCTAGGCACCAA CTCTTTGATGTCACGCACGATTTC
Elongation factor 1-α	*EF1-α*-F *EF1-α*-R	150 bp	56	63	1.87	EC959059	GAACTGGGTGCTTGATAGGC AACCAAAATATCCGGAGTAAAAGA

### Disease Assessment

The severity of downy mildew was evaluated two times, at 15 dps2 and 15 days post the third spray (dps3). Fifteen randomly selected leaves from each grapevine were evaluated for the severity of downy mildew using a six-degree scale based on the disease symptoms and leaf damage according to Townsend and Heuberger (1943), where 0 = no symptoms, 1 = 1–10%, 2 = 10–25%, 3 = 25–50%, 4 = 50–75%, and 5 = 75–100% infection. The disease severity (DS) was calculated using the following formula:


$$\text{DS}\left(\%\right)=\frac{\Sigma \,a\,b}{A\,K}$$

where *a* = number of diseased plants having the same severity grade, *b* = severity grade, *A* = total number of plants, and *K* = highest degree of infection. The reduction percentage in DS was also calculated. The resistance degree of grapevines to *P. viticola* was rated according to a five-degree scale of Organisation Internationale de la vigne et du vin (OIV), where 1 = very low, 3 = low, 5 = medium, 7 = high, and 9 = very high.

### Vegetative Growth Parameters

At 15 days post the fourth spray (dps4), the vegetative growth parameters of grapevines were evaluated in the non-fruiting shoots in response to the applied treatments. The evaluated parameters included average shoot length (cm) and leaf surface area (cm^2^). To measure leaf area, sixth and seventh leaves from the tip of the growing shoot were used (Montero et al., 2000).

### Yield and Its Components’ Parameters

At harvesting time (50 dps4), when the soluble solid content (SSC%) of berries juice reached about 16–17% in the control grapevines, a representative sample of six clusters/grapevine was weighted to determine the average cluster weight (g). The average yield/grapevine (kg) was calculated by multiplying the average cluster weight (g) by the number of clusters/grapevine. In addition, the weight of 100 berries (g), the size of 100 berries (cm^3^), cluster length and width (cm), and berry length and diameter (mm) were also determined.

### Biochemical Analyses

At 15 dps2, in the first season only, the grapevine leaves of each treatment were analyzed for the total photosynthetic pigments as described by Harborne (1984), total phenolic content according to the method described by Malick and Singh (1980), electrolyte leakage (%) according to Shi et al. (2006), the lipid peroxidation following the method of Heath and Packer (1968), the content of H_2_O_2_ according to the method of Aebi (1984), and the content of ascorbic acid according to Law et al. (1983). In addition, the activities of peroxidase (POD) and polyphenol oxidase (PPO) enzymes were determined according to Maxwell and Bateman (1967) and Galeazzi et al. (1981), respectively.

### Chemical Properties of Berries

Fifty days from the fourth spray, the clusters used in the determination of the yield and its components were used also to estimate the total soluble solid content (TSS, Brix) using a hand refractometer model Master T (ATAGO Co., Ltd., Tokyo, Japan). Total acidity (g tartaric acid/100 ml juice) was determined as described by AOAC (1980), then the (TSS/acidity) ratio was calculated. Total sugar content (%) was determined according to the method of Sadasivam and Manickam (1996), and the total carotenoids content of berry skin (mg/g fw) was also determined as described by Mackinney (1941).

### Ultrastructure Studies

For scanning electron microscopy (SEM) observations, grapevine leaves were sampled after the second spray. A piece (1 cm^2^) of each treatment was dehydrated using graded ethanol series (10–100%) each stage for 10 min, dried using a critical point dryer (TEC-030), and coated with gold using a sputter coater (FDU-010). SEM observations were performed using a scanning electron microscope (JEOL 100 CX-II ASID-4D, Tokyo, Japan). The average number of closed and opened stomata (field area = 0.195 mm^2^ at 250X magnification), stomatal area, and stomatal pore area (at 4,500X magnification) in the leaves of grapevines were determined.

For transmission electron microscopy (TEM) observations, grapevine leaves were sampled after the fourth spray. A grapevine leaf piece (1 cm^2^) from each treatment under 2.5% glutaraldehyde in 0.1 M sodium cacodylate buffer (pH = 7) was kept in the same fixative for 24 h at 20 ± 2°C. The specimen was dehydrated using 10 graded ethanol series (10–100%) each stage for 10 min. The specimen was then processed for embedding using ethanol and propylene oxide and embedded in gelatin capsules filled with fresh Araldite, and placed in an oven at 60°C for 60 h. Ultrathin sections were cut on Reichert Ultramicrotome using a glass knife, and the sections were picked up on a 200-mesh copper grid and stained with uranyl acetate followed by lead citrate. TEM observations were performed using a transmission electron microscope (JEM-1230; JEOL Ltd., Tokyo, Japan).

### Cytotoxicity and Genotoxicity Bioassays

For the evaluation of cytotoxicity, two cell lines were used; Vero (African green monkey kidney epithelial normal cells line) and BEAS-2B (human normal lung epithelial cells). To grow a complete cell monolayer, a 96-well-tissue-culture plate was inoculated with 10^5^ cells ml^–1^ (100 μl per well) and incubated at 37°C for 24 h. Twofold dilution of the tested sample was made in RPMI medium with 2% serum (maintenance medium). MTT standard cytotoxicity assay was utilized to investigate the cytotoxic effects of SiNPs at different concentrations (0, 25, 50, 100, 150, 200, 500, and 1,000 ppm), at 48-h exposure time, three times as described by Mosmann (1983). For each cell line, the cell viability and mortality percentages against each concentration and the half-maximal inhibitory concentration (*IC*50) were determined.

For the evaluation of genotoxicity, healthy onion bulbs were kept in glass flasks containing distilled water for growing their roots (5 days, 25 ± 2°C). The rooted bulbs were separately treated with an aqueous solution of SiNPs at 100 and 150 ppm for 24 h. Rooted bulbs treated only with distilled water for 24 h served as a negative control. After the exposure time, the root tips from each treatment were cut into 5 mm length, fixed in Carnoy’s solution (glacial acetic acid:ethanol 1:3 v/v) for 48 h at 4°C, and stained with aceto-orcein stain for 4 h according to the protocol of Kao (1975). The stained root tips were then examined using a compound microscope (100x objective lens) for cell scoring. For each treatment, about 2,000 cells (10 slides) were examined and photographed for any abnormality in the different stages of mitotic division. The mitotic index and mitotic phase index were calculated according to the following formulae:


$$\mathrm{Mitotic}\mathrm{phase}\mathrm{index}\left(\%\right)=\frac{\mathrm{Number}\,\mathrm{of}\,\mathrm{dividing}\,\mathrm{cells}\,\mathrm{of}\,\mathrm{phases}}{\mathrm{Total}\,\mathrm{number}\,\mathrm{of}\,\mathrm{dividing}\,\mathrm{cells}}\times \,100$$

$$\mathrm{Mitotic}\mathrm{index}\left(\%\right)=\frac{\mathrm{Total}\,\mathrm{number}\,\mathrm{of}\,\mathrm{dividing}\,\mathrm{cells}}{\mathrm{Total}\,\mathrm{number}\,\mathrm{of}\,\mathrm{dividing}\,\mathrm{cells}+\mathrm{non}\,\text{-}\,\mathrm{dividing}\,\mathrm{cells}}\times \,100$$

### Statistical Analyses

All analyses were carried out three times. The obtained results were analyzed using the statistical analysis software CoStat (version 6.4). Testing for the homogeneity of variances was performed using Bartlett’s test. Comparisons between means were analyzed using Duncan’s multiple range test at *p* ≤ 0.05 (Duncan, 1955).

## Results

### Time-Course Transcriptome Analysis of the Defense-Responsive Genes

**FIGURE 1:**
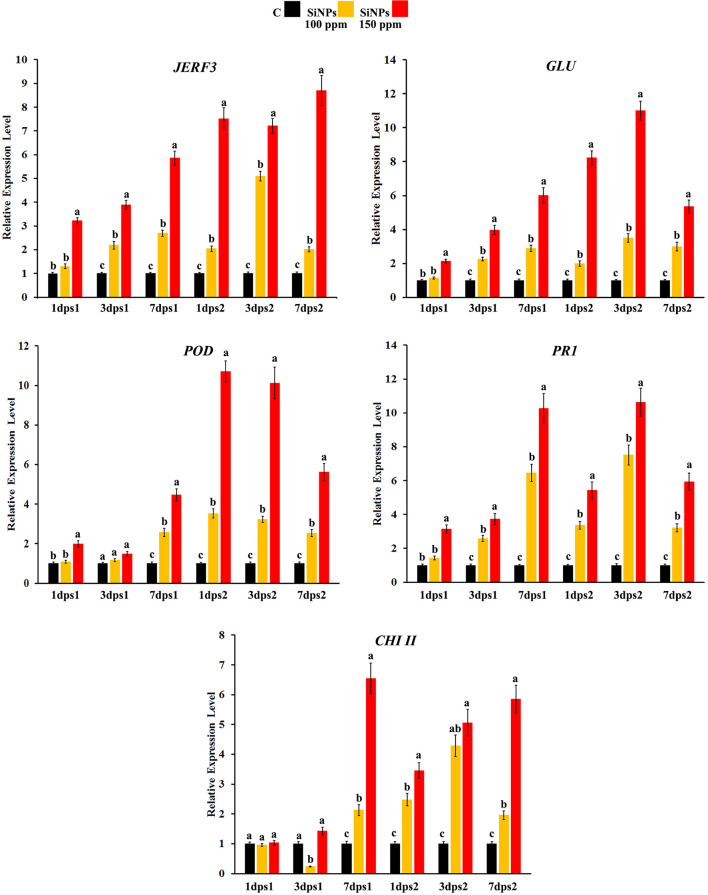
Histograms showing the relative transcriptional expression levels of the transcription factor jasmonate and ethylene-responsive factor 3 (*JERF3*) and four defense-related genes [β-1,3-glucanase (*GLU*), peroxidase (*POD*), pathogenesis-related-protein 1 (*PR1*), and chitinase class II (*CHI II*)] in the leaves of grapevines infected with downy mildew in response to spraying of silica nanoparticles (SiNPs) at 100 and 150 ppm after 1, 3, and 7 days post the first spray (dps1), and days post the second spray (dps2). Where C: untreated-infected with downy mildew, SiNPs at 100: infected with downy mildew and treated with SiNPs at 100 ppm, and SiNPs at 150: infected with downy mildew and treated with SiNPs at 150 ppm. In each time for each studied gene, columns superscripted with the same letter are not significantly different according to Duncan’s multiple range test (*p* ≤ 0.05). Each value represents the mean of three biological replicates; each sample was analyzed three times. Error bars represent SEs.

Changes over time in the expression profile of some defense-responsive genes were monitored in grapevine leaves infected with downy mildew at 1, 3, and 7 dps1, as well as 1, 3, and 7 dps2 with SiNPs at 0, 100, and 150 ppm (Figure 1). The study included the jasmonate and ethylene-responsive factor 3 (*JERF3*) and four defense-responsive genes, namely, *β*-1,3-glucanase (*GLU*), peroxidase (*POD*), pathogenesis-related-protein 1 (*PR1*), and chitinase class II (*CHI II*). For *JERF3*, the results obtained from qPCR revealed that spraying grapevines with SiNPs significantly provoked the transcriptional gene expression level, except at 1 dps1 for the 100 ppm concentration, in comparison with the unsprayed control grapevines. However, the triggering effect of the SiNPs treatment at 150 ppm was higher than that at 100 ppm at all the studied time points. Moreover, the inducing effect was due to a gradual elevation of both treatments over time after the first spray, except for SiNPs treatment at 100 ppm, where the gene expression reached its maximum level at 3 dps2 and then declined at 7 dps2. In this regard, the highest gene expression level was noticed for the SiNPs treatment at 150 ppm at 7 dps2 (8.7-fold), in comparison with the unsprayed-infected grapevines. Regarding *GLU*, spraying grapevines with SiNPs at both concentrations led to a considerable upregulation in the gene expression at all-time points, except for the SiNPs treatment at 100 ppm at 1 dps1. However, the inducing effect of the SiNPs treatment at 150 ppm was greater than that at 100 ppm at all-time points. After the first spray, the upregulation in the gene expression was increased over time, while after the second spray, the gene expression in the case of both treatments reached its maximum level at 3 dps2, and then declined at 7 dps2. The highest gene expression level was recorded for the SiNPs treatment at 150 ppm at 3 dps2 (11-fold). Regarding *POD*, no significant change was observed in the gene expression in response to spraying of SiNPs at 100 ppm at 1 and 3 dps1, in comparison with the unsprayed-infected grapevines while this treatment induced the gene expression at all the next time points. In contrast, grapevines sprayed with SiNPs at 150 ppm significantly enhanced the gene expression at all-time points. In general, the gene expression in response to both treatments was higher at the time points after the second spray those after the first one. In this regard, the highest gene expression level was observed for the SiNPs treatment at 150 ppm at 1 dps2 (10.7-fold), after which the gene expression decreased. For *PR1*, the obtained data exhibited an inducing effect for both the applied treatments, except for SiNPs at 100 ppm at 1 dps1, in comparison with the unsprayed-infected grapevines. The triggering effect of both treatments increased over the time after the first spray; however, an inducing effect of the SiNPs treatment at 150 ppm was higher than that at 100 ppm. After the second spray, the overexpression due to both treatments decreased at 1 dps2, elevated at 3 dps2 reaching their maximum level, and then declined at 7 dps2. The highest expression level for *PR1* was recorded for the SiNPs treatment at 150 ppm at 3 dps2 (10.6-fold). For *CHI II*, no change in the gene expression was observed for both treatments of SiNPs at 1 dps1. At 3 dps1, downregulation was observed in response to spraying with SiNPs at 100 ppm, while no change in the gene expression was recorded for the SiNPs treatment at 150 ppm in comparison with the unsprayed-infected grapevines. At 7 dps1, both treatments led to a significant upregulation of the gene expression. After the second spray, a considerable overexpression was observed for both treatments at all time points. However, the inducing effect of the SiNPs treatment at 150 ppm decreased at 1 dps2 than that at 7 dps1, and then re-elevated over the next time points, while the gene expression due to the SiNPs treatment at 100 ppm increased at 1 dps2 and 3 dps2 and then decreased at 7 dps2. The highest gene expression was noticed for the SiNPs treatment at 150 ppm at 7 dps1 (6.5-fold).

### Effect on the Disease Severity

The data presented in Table 2 show that spraying with SiNPs at different concentrations significantly reduced, at varying extents, DS in grapevines infected with downy mildew at both seasons, in comparison with the unsprayed-infected grapevines. However, a reduction in DS was considerably higher at 15 dps2 than that at 15 dps3. Compared with grapevines treated with the chemical anti-oomycete fungicide, the highest reduction in DS was observed for SiNPs treatment at 150 ppm, at 15 dps2, recording 78.2 and 81.5% reduction in seasons 2020 and 2021, respectively.

**TABLE 2:** Effects of foliar application of silica nanoparticles (SiNPs) on the disease severity (DS) of grapevines infected with downy mildew at 15 days post the second spray (dps2) and 15 days post the third spray (dps3).*

Treatment**		15 dps2						15 dps3					
		2020			2021			2020			2021		
		Disease severity (%)	Reduction (%)	Degree of resistance	Disease severity (%)	Reduction (%)	Degree of resistance	Disease severity (%)	Reduction (%)	Degree of resistance	Disease severity (%)	Reduction (%)	Degree of resistance
Control		20.9 ± 1.6^a^	0.0^e^	5	20.0 ± 1.3^a^	0.0^e^	5	81.6 ± 2.6^a^	0.0^e^	1	79.0 ± 1.9^a^	0.0^e^	1
Fungicide		2.0 ± 0.1^e^	90.4 ± 1.2^a^	9	1.8 ± 0.2^e^	91.0 ± 0.9^a^	9	14.9 ± 0.5^e^	81.7 ± 1.2^a^	7	12.4 ± 1.3^e^	84.3 ± 1.3^a^	7
SiNPs (ppm)	50	16.4 ± 0.9^b^	21.4 ± 1.7^d^	5	15.9 ± 0.4^b^	20.4 ± 1.1^d^	5	70.5 ± 1.6^b^	13.5 ± 2.5^d^	3	68.1 ± 1.2^b^	13.7 ± 1.6^d^	3
	100	11.3 ± 1.1^c^	46.1 ± 1.3^c^	7	10.8 ± 1.4^c^	45.5 ± 3.4^c^	7	50.6 ± 0.9^c^	37.9 ± 3.0^c^	5	47.9 ± 1.6^c^	39.3 ± 3.5^c^	5
	150	4.6 ± 0.8^d^	78.2 ± 2.1^b^	9	3.7 ± 0.4^d^	81.5 ± 5.7^b^	9	21.3 ± 1.0^d^	73.9 ± 1.9^b^	7	19.1 ± 1.1^d^	75.8 ± 1.6^b^	7

*Values are the means of 45 replicates (15 leaves per grapevine) ± SD. The resistance degree was rated according to Organisation Internationale de la vigne et du vin (OIV). **Values of each column followed by the same letter(s) are not significantly different according to Duncan’s multiple range test (*p* ≤ 0.05).

### Effect on the Vegetative Growth Parameters

The results obtained from the field experiment indicated that all SiNPs treatments enhance, at varying extents, the shoot length and leaf surface area of grapevines infected with downy mildew, compared with the unsprayed-infected grapevines at both seasons (Table 3). In this regard, the highest shoot length was noticed for SiNPs treatment at 150 ppm in seasons 2020 and 2021 recording 155.3 and 162 cm, respectively. At the same time, the highest leaf area was observed for SiNPs treatment at 150 ppm in seasons 2020 and 2021 recording 138, and 141.3 cm^2^, respectively.

**TABLE 3:** Effects of foliar application of SiNPs on vegetative growth parameters of grapevines infected with downy mildew at 15 days post the fourth spray.*

Treatment**		Shoot length (cm)		Leaf surface area (cm^2^)	
		
		2020	2021	2020	2021
Control		125.7 ± 7.4^b^	128.3 ± 5.7^c^	106.0 ± 12.2^c^	110.0 ± 8.7^c^
Fungicide		132.0 ± 10.6^b^	136.3 ± 10.4^bc^	112.0 ± 12.5^bc^	118.0 ± 8.7^bc^
SiNPs (ppm)	50	142.0 ± 8.7^ab^	148.0 ± 7.0^ab^	124.0 ± 14.0^abc^	128.0 ± 6.9^ab^
	100	150.0 ± 9.2^a^	158.3 ± 6.5^a^	134.3 ± 10.7^ab^	136.0 ± 7.2^a^
	150	155.3 ± 9.9^a^	162.0 ± 10.6^a^	138.0 ± 8.7^a^	141.3 ± 10.3^a^

*Values are the means of three replicates ± SD. **Values of each column followed by the same letter(s) are not significantly different according to Duncan’s multiple range test (*p* ≤ 0.05).

### Effect on the Yield and Its Components’ Parameters

The results presented in Table 4 show the effects of spraying of downy mildew- infected grapevines with SiNPs treatments on their yield parameters. All applied concentrations of SiNPs significantly improved the grapevine yield and its components at both seasons, in comparison with the unsprayed-infected grapevines. In this concern, SiNPs treatment at 150 ppm recorded the highest values at the two seasons regarding the yield per grapevine, cluster weight, the weight of 100 berries, the size of 100 berries, cluster length and width, and berry length and diameter.

**TABLE 4:** Effects of foliar application of SiNPs on the yield and its components of grapevines infected with downy mildew at 50 days from the fourth spray.*

Treatment**		Yield/vine (kg)		Cluster weight (g)		Weight of 100 berries (g)		Size of 100 berries (cm^3^)		Cluster length (cm)		Cluster width (cm)		Berry length (mm)		Berry diameter (mm)	
								
		2020	2021	2020	2021	2020	2021	2020	2021	2020	2021	2020	2021	2020	2021	2020	2021
Control		15.2 ± 0.7^d^	15.9 ± 0.6^c^	634.0 ± 27.5^d^	665.3 ± 25.3^c^	172 ± 7.2^c^	180 ± 9.2^c^	156 ± 9.1^d^	160 ± 12.5^c^	26.3 ± 0.6^c^	25.0 ± 0.5^c^	12.0 ± 1.0^c^	13.3 ± 1.2^c^	16.3 ± 0.6^b^	17.3 ± 1.2^d^	14.0 ± 1.7^c^	15.3 ± 1.5^c^
Fungicide		16.9 ± 0.8^c^	17.6 ± 0.7^b^	702.3 ± 34.6^c^	734.7 ± 28.1^b^	194 ± 14.4^b^	198 ± 15.9^bc^	174 ± 8.7^cd^	180 ± 15.1^bc^	30.0 ± 1.7^b^	30.0 ± 1.1^b^	14.3 ± 1.5^bc^	15.0 ± 1.0^bc^	17.0 ± 1.0^b^	17.7 ± 1.2^cd^	14.7 ± 0.5^bc^	16.7 ± 1.2^bc^
SiNPs (ppm)	50	17.4 ± 0.7^bc^	18.2 ± 0.6^ab^	725.0 ± 27.9^bc^	760.0 ± 26.2^ab^	204 ± 12.4^ab^	212 ± 18.3^ab^	186 ± 7.2^bc^	192 ± 15.9^ab^	32.0 ± 1.3^b^	33.0 ± 0.9^ab^	14.3 ± 1.2^bc^	16.0 ± 1.7^ab^	18.0 ± 1.7^ab^	20.0 ± 1.7^bc^	17.0 ± 1.0^ab^	18.0 ± 1.0^ab^
	100	18.4 ± 0.6^ab^	19.0 ± 0.8^a^	765.3 ± 25.2^ab^	792.0 ± 32.2^a^	218 ± 10.4^a^	228 ± 15.9^a^	204 ± 12.2^ab^	210 ± 14.0^a^	35.0 ± 1.0^a^	36.0 ± 1.0^a^	16.0 ± 1.0^ab^	18.0 ± 1.0^a^	19.7 ± 1.2^a^	22.0 ± 1.6^ab^	17.0 ± 1.2^ab^	19.0 ± 0.6^a^
	150	18.8 ± 0.6^a^	19.4 ± 0.6^a^	785.3 ± 22.0^a^	810.3 ± 23.0^a^	226 ± 12.5^a^	234 ± 12.1^a^	210 ± 12.0^a^	218 ± 11.1^a^	35.7 ± 1.2^a^	36.0 ± 1.2^a^	17.0 ± 1.5^a^	17.0 ± 0.5^ab^	20.0 ± 1.0^a^	23.0 ± 1.0^a^	18.0 ± 1.5^a^	19.3 ± 1.0^a^

*Values are the means of three replicates ± SD. **Values of each column followed by the same letter(s) are not significantly different according to Duncan’s multiple range test (*p* ≤ 0.05).

### Effect on the Total Photosynthetic Pigments

The obtained data presented in Table 5 exhibited that spraying of downy mildew-infected grapevines with SiNPs at the studied concentrations led to improving, at varying extents, of the contents of the photosynthetic pigments (Chl. *a*, Chl. *b*, and carotenoids), in comparison with the unsprayed-infected grapevines. In this regard, the highest content of the total photosynthetic pigments was recorded for grapevines sprayed with SiNPs at 150 ppm recording 2.28 mg g^–1^ fresh weight.

**TABLE 5:** Effects of foliar application of SiNPs on the total photosynthetic pigments in the leaves of grapevines infected with downy mildew at 15 days post the second spray.*

Treatment**		Chl. *a* (mg g^–1^ fresh weight)	Chl. *b* (mg g^–1^ fresh weight)	Carotenoids (mg g^–1^ fresh weight)	Total pigments (mg g^–1^ fresh weight)
Control		0.698 ± 0.07^cd^	0.503 ± 0.01^b^	0.368 ± 0.05^ab^	1.57 ± 0.12^c^
Fungicide		0.835 ± 0.06^b^	0.609 ± 0.03^a^	0.462 ± 0.05^a^	1.91 ± 0.08^b^
SiNPs (ppm)	50	0.626 ± 0.02^d^	0.462 ± 0.08^b^	0.271 ± 0.05^b^	1.36 ± 0.03^c^
	100	0.750 ± 0.04^bc^	0.466 ± 0.04^b^	0.353 ± 0.06^ab^	1.57 ± 0.11^c^
	150	1.160 ± 0.08^a^	0.691 ± 0.05^a^	0.429 ± 0.09^a^	2.28 ± 0.21^a^

*Values are the means of three replicates ± SD, SiNPs treatments were applied four times; 15, 45, 60, and 85 days from bud burst. **Values of each column followed by the same letter(s) are not significantly different according to Duncan’s multiple range test (*p* ≤ 0.05).

### Effect on the Total Phenolic Content and Activities of the Oxidative Enzymes Peroxidase and Polyphenol Oxidase

Table 6 represents the effects of spraying downy mildew-infected grapevines with SiNPs at 0, 50, 100, and 150 ppm on their total phenolic content and activities of POD and PPO enzymes. All applied SiNPs treatments significantly enhanced the total phenolic content and activities of both the studied enzymes in comparison with the unsprayed-infected grapevines. In this concern, the highest values were achieved by the SiNPs treatment at 150 ppm, compared with grapevines infected-treated with the chemical anti-oomycete fungicide, except for the POD enzyme where all treatments of SiNPs significantly induced their activity at the same extent.

**TABLE 6:** Effects of foliar application of SiNPs on the total phenolic content and activities of peroxidase and polyphenol oxidase enzymes in the leaves of grapevines infected with downy mildew at 15 days post the second spray.*

Treatment**		Total phenols (mg.g^–1^ fresh weight)	Peroxidase (ΔA_470_ min^–1^ g^–1^ fresh weight)	Polyphenol oxidase (ΔA_420_ min^–1^ g^–1^ fresh weight)
Control		719.5 ± 11.0^d^	3.7 ± 0.6^bc^	1.5 ± 0.2^d^
Fungicide		557.9 ± 17.4^e^	3.4 ± 0.1^c^	2.3 ± 0.4^cd^
SiNPs (ppm)	50	774.3 ± 8.7^c^	4.7 ± 0.8^ab^	2.9 ± 0.5^bc^
	100	976.3 ± 25.6^b^	5.3 ± 0.7^a^	3.3 ± 0.4^b^
	150	1243.2 ± 27.4^a^	5.3 ± 0.4^a^	4.2 ± 0.6^a^

*Values are the means of three replicates ± SD, SiNPs treatments were applied four times; 15, 45, 60, and 85 days from bud burst. **Values of each column followed by the same letter(s) are not significantly different according to Duncan’s multiple range test (*p* ≤ 0.05).

### Effect on the Electrolyte Leakage, Lipid Peroxidation, and Contents of Hydrogen Peroxide and Ascorbic Acid

The data presented in Table 7 show the effects of applying SiNPs treatments on grapevines infected with downy mildew on the electrolyte leakage, lipid peroxidation, and contents of hydrogen peroxide and ascorbic acid in their leaves. The obtained results indicated that applying SiNPs treatments leads to a considerable reduction in the electrolyte leakage, lipid peroxidation, and hydrogen peroxide content while enhancing the ascorbic acid content. In this regard, the best results were recorded for the SiNPs treatment at 150 ppm, in comparison with the infected grapevines treated with the chemical anti-oomycete fungicide.

**TABLE 7:** Effects of foliar application of SiNPs on the electrolyte leakage, lipid peroxidation, and contents of hydrogen peroxide and ascorbic acid in the leaves of grapevines infected with downy mildew at 15 days post the second spray.*

Treatment**		Electrolyte leakage (%)	Lipid peroxidation (mmol MDA.g^–1^ fresh weight)	Hydrogen peroxide (μmol.g^–1^ fresh weight)	Ascorbic acid (mg.g^–1^ dry weight)
Control		111.5 ± 2.2^a^	15.1 ± 0.05^a^	1.0 ± 0.09^a^	17.6 ± 0.3^b^
Fungicide		101.7 ± 2.6^b^	10.9 ± 0.31^c^	0.8 ± 0.02^b^	10.8 ± 1.5^c^
SiNPs (ppm)	50	115.1 ± 2.7^a^	11.4 ± 0.08^b^	0.7 ± 0.03^b^	18.5 ± 0.6^b^
	100	101.9 ± 2.8^b^	10.2 ± 0.16^d^	0.5 ± 0.06^c^	20.3 ± 0.4^a^
	150	97.7 ± 2.2^b^	10.1 ± 0.08^d^	0.4 ± 0.04^c^	20.8 ± 0.5^a^

*Values are the means of three replicates ± SD, SiNPs treatments were applied four times; 15, 45, 60, and 85 days from bud burst. **Values of each column followed by the same letter(s) are not significantly different according to Duncan’s multiple range test (*p* ≤ 0.05).

### Effect on the Chemical Properties of Berries

The data presented in Table 8 indicate the effects of the foliar application of SiNPs treatments on the chemical properties of berries of grapevines infected with downy mildew. For the two seasons, all treatments significantly improved, to varying extents, the TSS content, total acidity, TSS/acidity ratio, total sugar content, and total carotenoid content of berry skin, in comparison with the unsprayed-infected grapevines. The highest values were mostly recorded for grapevines sprayed with SiNPs at 100 and 150 ppm in comparison with the infected grapevines treated with the chemical anti-oomycete fungicide.

**TABLE 8:** Effects of foliar application of SiNPs on the chemical properties of berries of grapevines infected with downy mildew at 50 days from the fourth spray.*

Treatment**		Soluble solids content (TSS) (°Brix)		Total acidity (g tartaric acid/100 mL juice)		TSS/Acidity		Total sugars content (%)		Total carotenoids content of berry skin (mg/g fw)	
					
		2020	2021	2020	2021	2020	2021	2020	2021	2020	2021
Control		16.6 ± 0.53^c^	17.0 ± 0.61^b^	0.584 ± 0.02^a^	0.568 ± 0.01^a^	28.5 ± 2.1^c^	30.0 ± 2.1^b^	15.1 ± 0.48^c^	15.3 ± 0.25^c^	2.9 ± 0.50^c^	3.4 ± 0.15^d^
Fungicide		17.2 ± 0.53^bc^	17.4 ± 0.52^b^	0.558 ± 0.03^a^	0.538 ± 0.03^a^	30.9 ± 2.3^bc^	32.4 ± 2.4^b^	15.4 ± 0.52^bc^	15.6 ± 0.32^c^	3.3 ± 0.51^c^	4.1 ± 0.16^c^
SiNPs (ppm)	50	18.0 ± 0.62^ab^	18.4 ± 0.34^a^	0.512 ± 0.03^b^	0.502 ± 0.02^b^	35.2 ± 2.4^ab^	36.7 ± 1.9^a^	16.0 ± 0.33^ab^	16.3 ± 0.18^b^	4.3 ± 0.48^b^	4.9 ± 0.45^b^
	100	18.4 ± 0.40^a^	18.8 ± 0.42^a^	0.496 ± 0.02^b^	0.488 ± 0.02^b^	37.2 ± 2.6^a^	38.6 ± 1.9^a^	16.2 ± 0.42^ab^	16.6 ± 0.26^ab^	4.9 ± 0.52^ab^	5.3 ± 0.36^ab^
	150	18.8 ± 0.56^a^	19.0 ± 0.23^a^	0.483 ± 0.02^b^	0.472 ± 0.01^b^	39.0 ± 2.8^a^	39.8 ± 1.0^a^	16.3 ± 0.46^a^	16.7 ± 0.20^a^	5.3 ± 0.46^a^	5.5 ± 0.26^a^

*Values are the means of three replicates ± SD. **Values of each column followed by the same letter(s) are not significantly different according to Duncan’s multiple range test (*p* ≤ 0.05).

### Electron Microscopic Observations

**FIGURE 2:**
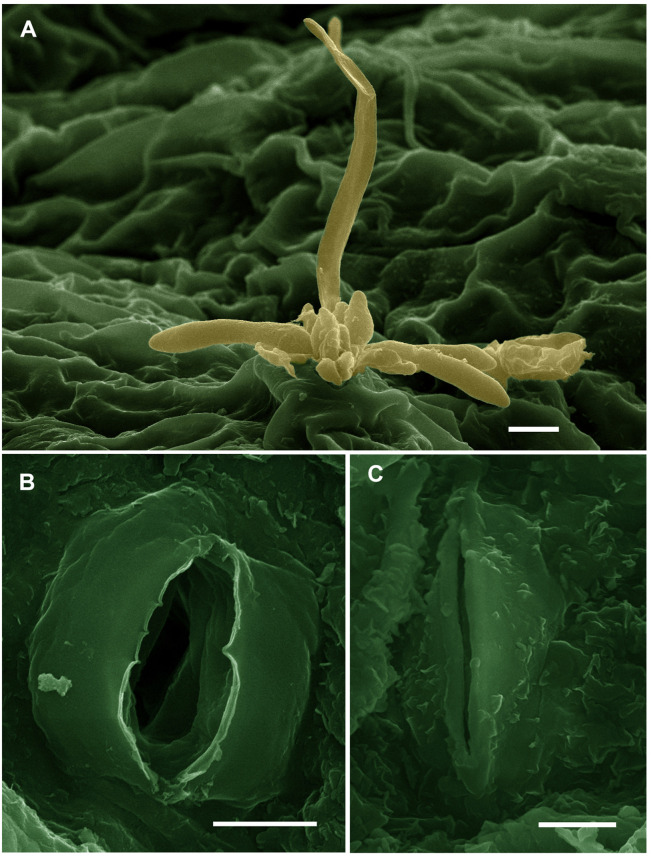
Scanning electron micrographs showing **(A)** sporangiophores of *Plasmopara viticola* emerging from the stomatal pore on the abaxial surface of a grapevine leaf (bar = 10 μm), **(B)** opened stomata in a leaf from the untreated control grapevine (bar = 5 μm), and **(C)** closed stomata in a grapevine leaf treated with SiNPs at 150 ppm (bar = 5 μm). These micrographs were colorized using the Adobe Photoshop CS6 software.

The results obtained from SEM on the number of closed and opened stomata, stomatal area, and stomatal pore area in grapevine leaves in response to spraying with SiNPs at 150 ppm are presented in Table 9. It was found that the treatment of downy mildew-infected grapevines with SiNPs at 150 ppm led to a significant increment in the number of closed stomata and decreased the number of opened stomata when compared to the untreated control grapevines. Moreover, the average stomatal area and stomatal pore area were also significantly reduced in response to this treatment, compared with the untreated control grapevines. SEM observations on the abaxial surface of a grapevine leaf infected with downy mildew showed the sporangiophores of *P. viticola* emerging from the stomatal pore of an untreated grapevine leaf (Figure 2A), opened stomata from an untreated grapevine leaf (Figure 2B), and closed stomata from a grapevine leaf treated with SiNPs at 150 ppm (Figure 2C).

**TABLE 9:** Scanning electron microscopic observations on the average number of closed and opened stomata (field area = 0.195 mm^2^ at 250X magnification), stomatal area, and stomatal pore area (at 4500X magnification) in the leaves of grapevines infected with downy mildew in response to foliar application of SiNPs at 150 ppm.*

Treatment**	Stomatal number		Stomatal area (μm^2^)	Stomatal pore area (μm^2^)
	opened	Closed		
Control	23.3 ± 2.5^a^	12.7 ± 3.1^b^	247.4 ± 33.7^a^	120.6 ± 14.4^a^
SiNPs	9.3 ± 1.9^b^	23.7 ± 4.2^a^	88.2 ± 10.2^b^	54.1 ± 9.6^b^

*Values are the means of three replicates ± SD, SiNPs treatments were applied four times; 15, 45, 60, and 85 days from bud burst. **Values of each column followed by the same letter(s) are not significantly different according to Duncan’s multiple range test (*p* ≤ 0.05).

**FIGURE 3:**
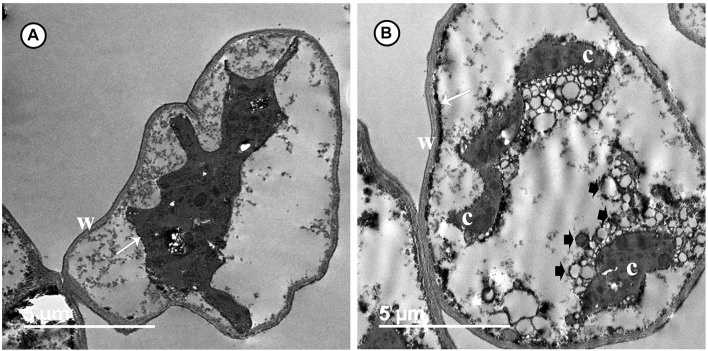
Transmission electron micrographs of grapevine leaf cells infected with *P. viticola*. Where **(A)** the cell is completely plasmolized. Note the thick wall (W) and cell membrane (arrow) moved away from the cell wall, and **(B)** the cell showing a disruption of their components. Note a thick and an electron-dense cell membrane (arrow). Note also abnormal chloroplasts (C), numerous granules (arrowheads), and a thick wall (W). Bar = 5.0 μm.

**FIGURE 4:**
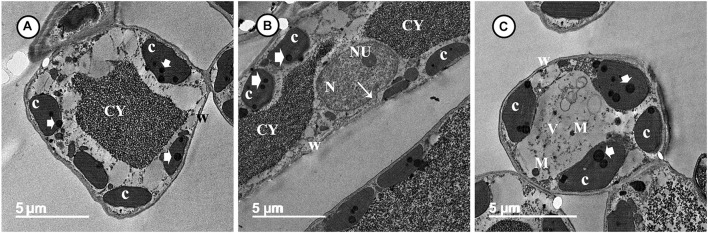
Transmission electron micrographs of grapevine leaf cells infected with *P. viticola* after the treatment by SiNPs. Where **(A)** A normal grapevine cell showing normal chloroplasts (C) with electron-dense plastoglobuli (arrowheads). Note a normal thin wall (W) and granulated cytoplasm (CY). Bar = 5.0 μm. **(B)** A normal grapevine cell showing normal chloroplasts (C) with electron-dense plastoglobuli (arrowheads). Note a normal thin wall (W) and granulated cytoplasm (CY). Note also a normal nucleus (N) with nucleolus (NU) surrounded by a nuclear envelope (arrow). Bar = 5.0 μm, and **(C)** A normal grapevine cell showing normal chloroplasts (C) associated with the cell wall (W) and contain electron-dense plastoglobuli (arrowheads). Note unknown electron-dense materials (M) located in cell vacuole (V). Bar = 5.0 μm.

Transmission electron microscopy observations revealed considerable ultrastructural alterations in the cells of a grapevine leaf as a response to infection with *P. viticola.* These alterations included complete plasmolysis and disruption of the cellular components, abnormal chloroplasts, and thickening of the cell wall and cell membrane (Figures 3A,B). In contrast, observations on leaf cells of the infected grapevine sprayed with SiNPs at 150 ppm showed a normal ultrastructure, including normal thin cell wall, normal chloroplasts with electron-dense plastoglobuli, a normal nucleus with nucleolus surrounded by a nuclear envelope, and a granulated cytoplasm (Figures 4A–C).

### Cytotoxicity Evaluation of Silica Nanoparticles

**FIGURE 5:**
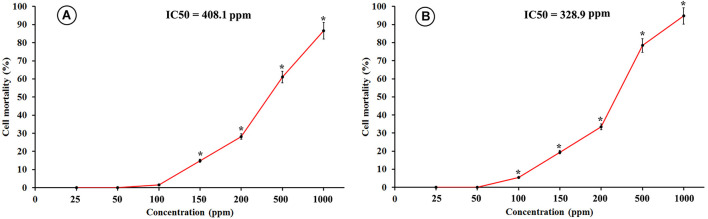
Cytotoxicity of SiNPs at exposure time of 48 h on **(A)** kidney epithelial cells (Vero), and **(B)** lung epithelial cells (BEAS-2B). Values represent the means of three replicates ± SE. *IC*50 = the half-maximal inhibitory concentration. ^∗^ = significant at *p* ≤ 0.05 compared with the untreated control.

The results obtained from the cytotoxicity assay indicated that the human lung epithelial cells (BEAS-2B) were more sensitive toward the toxicity of SiNPs compared to the kidney epithelial cells (Vero). SiNPs exhibited a concentration-dependent cytotoxic effect on both the utilized cell lines. For the Vero cell line, the obtained data revealed that SiNPs had no significant toxicity at concentrations ≤100 ppm. After which, the toxic effect was significantly increased with an increment in the SiNP concentration reaching its maximum point at 1,000 ppm, recording 86.5% mortality (Figure 5A). The cytotoxicity of SiNPs at 150 ppm was 14.9% while their *IC*50 value was recorded at 408.1 ppm. For the BEAS-2B cell line, the results showed that SiNPs had no significant toxicity at concentrations ≤50 ppm while their toxic effect was elevated with an increase in the SiNPs concentration after this point, reaching its maximum peak at 1,000 ppm, recording 94.8% mortality (Figure 5B). The cytotoxicity of SiNPs at 150 ppm was 19.5% while their *IC*50 value was recorded at 328.9 ppm.

### Genotoxicity Evaluation of Silica Nanoparticles

**FIGURE 6:**
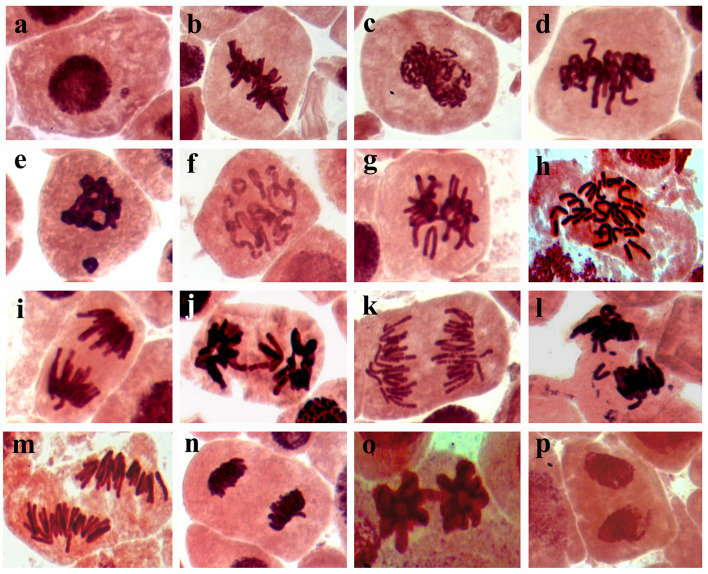
Light micrographs (1,000x) showing mitotic abnormalities in *Allium cepa* root tips in response to treating with SiNPs at 150 ppm for 24 h, where **(a)** micronucleus at interphase, **(b)** oblique at metaphase, **(c)** stickiness at metaphase, **(d)** disturbance at metaphase, **(e)** micronucleus at metaphase, **(f)** ring at metaphase, **(g)** non-congressing at metaphase, **(h)** polyploidy at anaphase, **(i)** late separation at anaphase, **(j)** bridge at metaphase, **(k)** disturbance at anaphase, **(l)** laggard at anaphase, **(m)** diagonal at anaphase, **(n)** disturbance at telophase, **(O)** bridge at telophase, and **(p)** diagonal at telophase.

The results obtained from the genotoxicity bioassay on the mitotic index, mitotic phase index, and total abnormality in the meristematic cells of onion (*Allium cepa* L.) roots singly exposed to SiNPs at 100 and 150 ppm for 24 h are presented in Table 10. A significant increase in the mitotic index was noticed in the onion root tips treated with SiNPs at 100 ppm. While no significant difference was recorded in this parameter for those treated with SiNPs at 150 ppm in comparison with the control treatment. However, various abnormalities were monitored at different mitotic phases as well as the interphase for both treatments. In addition, a significant total mitotic abnormality was observed in onion root tips in response to exposure to SiNPs recording, 23.4 and 29.1% for the treatments at 100 and 150 ppm, respectively. In this regard, the light microscopy observations showed various nuclear abnormalities in all mitotic phases in response to the treatment with SiNPs, especially at 150 ppm, including chromosome stickiness, disturbance, late separation, non-congression, bridge, laggards, formation of micronucleus, ring chromosome, polyploidy, and diagonal forms (Figure 6).

**TABLE 10:** Mitotic index, mitotic phase index, and total abnormality in the meristematic cells of *Allium cepa* root treated with SiNPs at 100 and 150 ppm for 24 h.^∙^

Treatment		Mitotic index (%)	Mitotic phase index (%)								Total abnormality (%)	
			Prophase		Metaphase		Anaphase		Telophase		Interphase	Mitotic
			Mitotic	Abn.	Mitotic	Abn.	Mitotic	Abn.	Mitotic	Abn.		
Control		5.63 ± 0.44	34.8	0	35.0	7.7	14.7	4.7	15.5	0.9	0.06 ± 0.03	13.3 ± 3.1
SiNPs (ppm)	100	8.03 ± 0.36*	22.2	0	40.6	12.9	24.6	6.8	12.6	3.7	0.15 ± 0.07*	23.4 ± 2.0*
	150	5.33 ± 0.27^ns^	33.3	4.1	37.9	11.5	16.7	10.1	12.1	3.5	0.10 ± 0.04*	29.2 ± 1.9*

^∙^Data obtained from 5,000 examined cells, Abn. = abnormal. * = significant difference and ns = not significant at *p* ≤ 0.05.

## Discussion

Silicon is classified as a functional plant nutrient and contributed to the improvement of plant development and production against many biotic and abiotic stresses (Luyckx et al., 2017b). One of the most important benefits of Si for plants is its role in triggering plant resistance against different fungal and oomycete diseases (Vivancos et al., 2015; Jadhao and Rout, 2020; Kaur et al., 2021). In addition, it promotes plant growth, yield, and fruit physicochemical properties (Farouk et al., 2017). In this study, we aimed to evaluate the field application of Si (in nanoscale) on the grapevine resistance to downy mildew at pathological, plant production, molecular, biochemical, and ultrastructural levels. In addition, their cytotoxic and genotoxic impacts were also assessed.

Time-course changes in the gene expression of some defense-responsive genes were monitored in the grapevine leaves infected with downy mildew and sprayed with SiNPs at 100 and 150 ppm. The results obtained from the qPCR revealed that spraying grapevines with SiNPs at both concentrations significantly provoked the transcriptional gene expression level of all investigated genes (*JERF3*, *GLU*, *POD*, *PR1*, and *CHI II*), at varying extents, indicating their contribution to the Si-induced immune responses in grapevines. *JERF3* regulates various defense-related genes in the plant activating their immune responses to a subset of invading pathogens (Gutterson and Reuber, 2004). This result is in agreement with that of Ghareeb et al. (2011) who reported an upregulation in the *JERF3* expression, which mediated the Si-induced immune responses in tomatoes, priming their resistance against bacterial wilt disease. Moreover, they also reported an overexpression in the *POD*, which is an oxidative stress marker gene, indicating that the reactive oxygen species (ROS) signaling pathways may contribute also to the Si-induced resistance in tomatoes. This result is in compliance with that obtained in this study on the overexpression of *POD* in grapevines treated with SiNPs. *POD* is an oxidoreductase gene, which has multiple physiological functions such as lignin polymerization, defense against ROS, and free radicals resulted from the infection (Shigeto and Tsutsumi, 2016; Rashad et al., 2020). This probable antioxidant mechanism is supported by the results obtained in this study of the elevated activities of POD and PPO enzymes and the reduced lipid peroxidation, hydrogen peroxide content, and the cellular electrolyte leakage as a response to spraying grapevines with SiNPs. In addition, an increment in the ascorbic acid content, which is a non-enzymatic antioxidant compound, had also been observed. The upregulation of *JERF3* triggers multiple defense-related genes in the ethylene-jasmonate signaling pathway priming the plant resistance against fungal/oomycete infections (Ku et al., 2018). In this regard, the results obtained from this study revealed also overexpression of the pathogenesis-related genes *GLU*, *PR1*, and *CHI II*. *GLU* is a pathogenesis-related gene, which belongs to family 2 encoding the *GLU* enzyme catalyzing the breakdown of 1,3-glucan molecules, which mainly constitute the cell wall of the oomycete pathogens (Funnell et al., 2004). While *CHI* encodes the chitinase enzyme catalyzing the breakdown of the *β*-1,4 bonds in the chitin molecules, which slightly constitute the oomycete cell wall (Ilham et al., 2008). *PR1* is an antifungal/anti-oomycete gene, which is involved in the plant immune responses against the attack of many phytopathogenic fungi and oomycetes (Sarowar et al., 2005; Breen et al., 2017). The overexpression of these defense-related genes may discuss a considerable reduction in the severity of downy mildew in grapevine leaves, which was reported in this study as a response to SiNPs application. The accumulation of anti-oomycete substances in the infected plant tissues is another proposed defensive mechanism, which mediated Si-induced immune responses. This probable mechanism is supported by an increment in the total phenolic compounds reported in this study. These reported results are in agreement with those reported by Farouk et al. (2017) on Roumy Ahmar grapevines infected with downy mildew.

One of the most important protective mechanisms reported in this study *via* SEM observations is an inducing effect of SiNPs on the stomatal closure, stomatal area, and stomatal pore area. In addition to their important roles in improving the plant growth and photosynthetic performance, and reducing the leaf transpiration rate to tolerate the drought stress (Verma et al., 2020b), this mechanism is a highly effective strategy for controlling the downy mildew disease because the stomata are the only gate for penetration and sporulation of *P. viticola*. Where their zoospores are attracted toward the stomata *via* the chemotactism to penetrate the plant tissue (Allègre et al., 2007). So, the stomatal closing effect of SiNPs reduces the penetration sites and the subsequent sporulation.

The data obtained from this study revealed that the application of SiNPs significantly enhanced the growth and yield parameters of grapevines infected with downy mildew as well as improved the quality of their berries. This result is in agreement with that reported by Schabl et al. (2020) on grapevines. Si spraying is one of the known forms of foliar fertilization, which improves the nutrients balance and physiological performance in the plant leading to enhance its growth, resistance, and productivity, and subsequently increase its yield (Laane, 2018). In the EU, Si products are classified as biostimulants rather than biofertilizers. Si constitutes one of the important nutrient components of plant cells. A number of functions are recognized for Si in plants, including the improvement of the plant structural strength, stimulation of many plant physiological processes, enhancement of the plant photosynthesis and nutrient uptake, promotion of plant growth and development, induction of plant resistance to biotic and abiotic stresses, regulation of the plant transpiration, and deterrent to herbivory (Luyckx et al., 2017a; Deshmukh et al., 2017; Laane, 2018). These reports are in compliance with the results obtained in this study on the enhancement of the total photosynthetic pigments and the improvement of the growth and yield parameters. Previous studies indicated that Si application can induce photosynthesis *via* improving light interception and transmission, in addition to reducing plant transpiration. The deposition of Si in the leaf blade keeps it erect, and in turn enhances the light interception (Verma et al., 2020a). Moreover, Si application can enhance photosynthesis *via* enlarging the chloroplast size and increasing the number of grana (Xie et al., 2014). This information is supported by the results obtained from TEM observations in this study. The infection of grapevines with downy mildew leads to a reduction in the photosynthetic pigments and diffusion of CO_2_ in the mesophyll tissue, and deformation of the chloroplast ultrastructure, which in turn leads to a decline in the photosynthesis process (Nogueira Júnior et al., 2019). This information was supported by the distortion observed, from the TEM observations, in the ultrastructure of the chloroplasts in the untreated-infected grapevine leaf and the elevation in the photosynthetic pigments associated with a decline in the DS reported in this study. Moreover, TEM observations indicated the positive effects of spraying SiNPs on the ultrastructure of a grapevine leaf in spite of the infection with downy mildew, showing their inducing effect on the plant resistance against *P. viticola*.

One of the interesting results obtained in this investigation is the considerable, concentration-dependent, cytotoxic, and genotoxic effects of both SiNPs treatments. This result is consistent with the finding of Aydin et al. (2017) who reported an inducing effect of SiNPs for cytotoxicity in a dose-dependent manner. Moreover, genetic changes due to apoptosis and/or DNA damage or repair were also reported. However, the cytotoxicity and genotoxicity of the NPs depend on many factors, including their size, shape, concentration, and dose (Dong et al., 2020). In a size-dependent study, Sahu et al. (2016) found that SiO_2_ particles have a cytotoxicity effect in their nanosize higher than in the micron-size in two types of cell lines. Nano-sized particles possess novel and effective physicochemical characteristics compared to their micron-sized ones such as the elevated surface/volume ratio and the increased surface reactivity (Napierska et al., 2010). The attractive properties of NPs enabled them to be a promising candidate to make breakthroughs in the fourth industrial revolution. In contrast, the extremely small size of the NPs enabled them to penetrate some cellular membranes and enter new sites, which are not accessible for micron-size particles. For example, the extremely small size of NPs enables them to pass across the blood-brain barrier. This cellular accessibility led the NPs to bind and affect the nuclear materials and other sensitive organelles in the cell resulting in cytotoxicity and genotoxicity (Murugadoss et al., 2017). So that, investigating the cytotoxic and genotoxic effects of NPs, at the studied dose/concentration, should be considered in all the investigations that deal with the biological applications of NPs, especially medicinal, agricultural, food, and veterinarian uses. In conclusion, the results obtained from this study revealed that spraying of downy mildew-infected grapevines with SiNPs at 150 ppm significantly induced the plant resistance, reduced the DS, improved the plant growth, and yield, as well as enhanced the berries quality. In contrast, the study also showed that this treatment had considerable cytotoxic and genotoxic effects at this direct dose/concentration. So, additional investigations to determine the SiNP residue in the produced edible plant parts are urgently needed. In addition, the pre-harvest interval, toxicity index, and risk assessment should be evaluated before any recommendation for use.

## Data Availability Statement

The raw data supporting the conclusions of this article will be made available by the authors, without undue reservation.

## Author Contributions

YR contributed to the idea and the design of the work, molecular investigation, SEM and TEM investigations, cytotoxicity and genotoxicity assays, statistical analyses of data, and manuscript and photos editing. HE-S contributed to the idea and design of the work, disease assessment, biochemical analysis, and SEM and TEM investigations. BB contributed to the field experiment, growth and yield evaluation, and analysis of the chemical properties of berries. ER contributed to the cytotoxicity and genotoxicity assays. DG contributed to the biochemical analysis and SEM investigation. All authors revised and approved the final manuscript.

## Conflict of Interest

The authors declare that the research was conducted in the absence of any commercial or financial relationships that could be construed as a potential conflict of interest.

## Publisher’s Note

All claims expressed in this article are solely those of the authors and do not necessarily represent those of their affiliated organizations, or those of the publisher, the editors and the reviewers. Any product that may be evaluated in this article, or claim that may be made by its manufacturer, is not guaranteed or endorsed by the publisher.
