# Supplementary figures and images for: Silica Nanoparticles as a Probable Anti-Oomycete Compound Against Downy Mildew, and Yield and Quality Enhancer in Grapevines: Field Evaluation, Molecular, Physiological, Ultrastructural, and Toxicity Investigations
Source: Front Plant Sci. 2021 Oct 28;12:763365. doi: 10.3389/fpls.2021.763365 (PMC8581734; doi:10.3389/fpls.2021.763365)

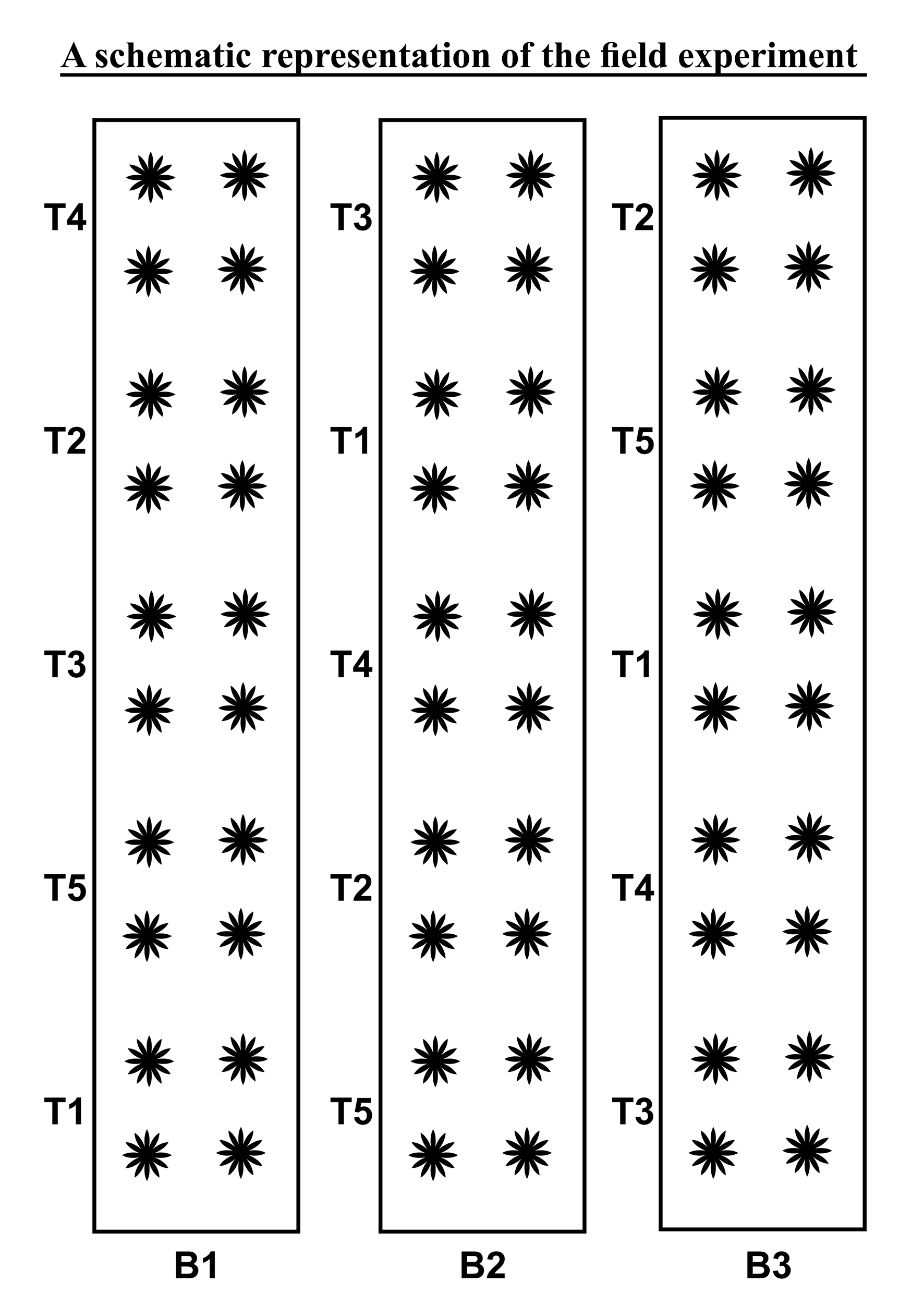

Supplement: Supplementary file 1 [file Image_1.JPEG]

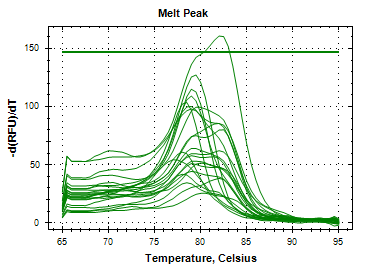


***JERF3***


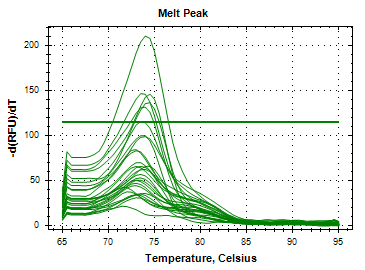


***PR1***


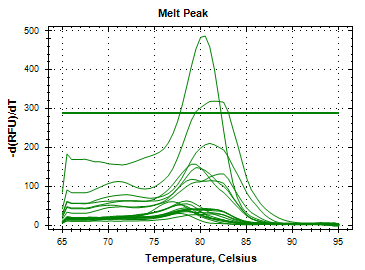


***CHI***


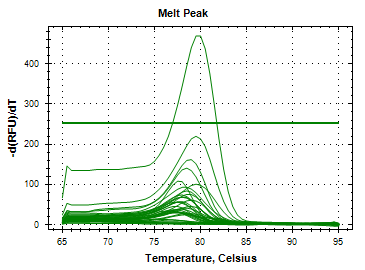


***Glu***


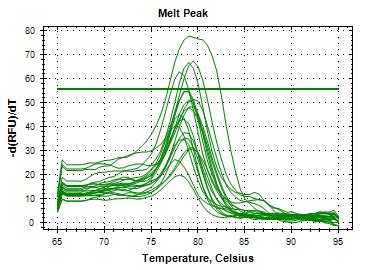


***POD***

Supplement: Supplementary file 2 [file Data_Sheet_1.doc]
