# Supplementary material for: Silica Nanoparticles as a Probable Anti-Oomycete Compound Against Downy Mildew, and Yield and Quality Enhancer in Grapevines: Field Evaluation, Molecular, Physiological, Ultrastructural, and Toxicity Investigations
Source: Front Plant Sci. 2021 Oct 28;12:763365. doi: 10.3389/fpls.2021.763365 (PMC8581734; doi:10.3389/fpls.2021.763365)
Supplement: Supplementary file 3 [file Table_1.docx]

The phenological growth stages of grapes according to BBCH scale (Hellman, 2003)

| **Principal growth stage** | **Code** | **Description** |
| --- | --- | --- |
| 0: Sprouting/Bud development |  |  |
|  | 00 | Dormancy: winter buds pointed to rounded, light or dark brown according to cultivar; bud scales more or less closed according to cultivar |
|  | 01 | Beginning of bud swelling: buds begin to expand inside the bud scales |
|  | 03 | End of bud swelling: buds swollen, but not green |
|  | 05 | “Wool stage”: brown wool clearly visible |
|  | 07 | Beginning of bud burst: green shoot tips just visible |
|  | 09 | Bud burst: green shoot tips clearly visible |
| 1: Leaf development | 11 | First leaf unfolded and spread away from shoot |
|  | 12 | 2nd leaves unfolded |
|  | 13 | 3rd leaves unfolded |
|  | 14 | Stages continuous till ... |
|  | 19 | 9 or more leaves unfolded |
| 5: Inflorescence emerge | 53 | Inflorescences clearly visible |
|  | 55 | Inflorescences swelling, flowers closely pressed together |
|  | 57 | Inflorescences fully developed; flowers separating |
| 6: Flowering | 60 | First flowerhoods detached from the receptacle |
|  | 61 | Beginning of flowering: 10% of flowerhoods fallen |
|  | 62 | 20% of flowerhoods fallen |
|  | 63 | Early flowering: 30% of flowerhoods fallen |
|  | 64 | 40% of flowerhoods fallen |
|  | 65 | Full flowering: 50% of flowerhoods fallen |
|  | 66 | 60% of flowerhoods fallen |
|  | 67 | 70% of flowerhoods fallen |
|  | 68 | 80% of flowerhoods fallen |
|  | 69 | End of flowering |
| 7: Development of fruits | 71 | Fruit set: young fruits begin to swell, remains of flowers lost |
|  | 73 | Berries groat-sized, bunches begin to hang |
|  | 75 | Berries pea-sized, bunches hang |
|  | 77 | Berries beginning to touch |
|  | 79 | Majority of berries touching |
| 8: Ripening of berries | 81 | Beginning of ripening: berries begin to develop variety-specific colour |
|  | 83 | Berries developing colour |
|  | 85 | Softening of berries |
|  | 89 | Berries ripe for harvest |
| 9: Senescence | 91 | After harvest; end of wood maturation |
|  | 92 | Beginning of leaf discolouration |
|  | 93 | Beginning of leaf-fall |
|  | 95 | 50% of leaves fallen |
|  | 97 | End of leaf-fall |
|  | 99 | Harvested product |

Hellman, Edward W. 2003. Grapevine Structure and Function. In: Oregon Viticulture (Edward W. Hellman, ed.), Corvallis, Oregon: Oregon State University Press.
